# Occurrence of antimicrobial-resistant bovine mastitis bacteria in Sakon Nakhon, Thailand

**DOI:** 10.14202/vetworld.2024.1202-1209

**Published:** 2024-06-02

**Authors:** Apinya Camsing, Nattamol Phetburom, Peechanika Chopjitt, Benjamabhorn Pumhirunroj, Patinya Patikae, Nattaya Watwiengkam, Suganya Yongkiettrakul, Anusak Kerdsin, Parichart Boueroy

**Affiliations:** 1 Department of Community Health, Faculty of Public Health, Kasetsart University, Chalermphrakiat Sakon Nakhon Province Campus, Sakon Nakhon 47000, Thailand; 2 Program in Animal Science, Faculty of Agricultural Technology, Sakon Nakhon Rajabhat University, Sakon Nakhon 47000, Thailand; 3 Faculty of Veterinary Sciences, Mahasarakham University, Mahasarakham 44000, Thailand; 4 National Center for Genetic Engineering and Biotechnology, National Science and Technology Development Agency, Khlong Nueng, Pathum Thani 12120, Thailand

**Keywords:** antimicrobial resistance, bovine mastitis, genotype, phenotype, Thailand

## Abstract

**Background and Aim:**

Bovine mastitis is an inflammation of the mammary gland of dairy cattle that causes economic losses due to poor quantity and quality of milk. The extensive or incorrect use of antibiotics has increased in the veterinary field, leading to the emergence of antibiotic-resistant pathogens worldwide. This study aimed to investigate bovine mastitis bacterial pathogens in Sakon Nakhon, Thailand.

**Materials and Methods:**

A total of 35 dairy farms were screened for clinical and subclinical mastitis using the California Mastitis Test and clinical examination. Polymerase chain reaction was used to characterize bacterial species-induced mastitis (380 isolates) in cattle and antimicrobial resistance genes, and disk diffusion and broth microdilution were used to characterize antimicrobial susceptibility.

**Results:**

The prevalence of *Staphylococcus epidermidis* (38.10%; 32/84)-induced mastitis in cattle was considerably high, followed by *Streptococcus agalactiae* (33.33%), *Streptococcus uberis* (25%), *Klebsiella pneumoniae* (8.33%), and *Staphylococcus aureus* (4.76%). In this study, *Staphylococcus* spp. isolates demonstrated 100% susceptibility to cefoxitin, and no antibiotic-resistance genes were identified. Tetracycline (TET) and macrolide-resistant genes of *Streptococcus* spp. revealed that *tet*M was predominant in 55.63% (79/142), followed by *tet*S + *erm*(B) (16.90%). Antibiotic susceptibility tests revealed the following resistance profiles to bacterial species: TET (85.92%), clindamycin (29.58%), erythromycin (15.49%), levofloxacin (14.08%), and penicillin (0%). Gram-negative bacterial isolates (*K. pneumoniae* [8.33%], *Klebsiella variicola* [2.38%], *Klebsiella quasipneumoniae* [1.19%], and *Escherichia coli* [1.19%]) were recovered and still susceptible to meropenem (100%), ceftazidime (97.06%), ceftriaxone (79.41%), and ciprofloxacin (79.41%).

**Conclusion:**

This result suggested that mastitis pathogens in this area were susceptible to most antimicrobials, with the exception of streptococci against TET. In this study, limited data were available including one from small-holder dairy farms and study only dairy farms in Sakon Nakhon, Thailand. So, more farms should be included in the future studies.

## Introduction

Bovine mastitis is an inflammation of the mammary gland caused by a complex interaction among three major factors: the host, infectious agent, and environment [1]. Mammary tissue damage during bovine mastitis results in a 70% decrease in the total loss of milk production [2]. It causes an economic loss by a decline in milk quality and quantity, adversely affecting animal health and welfare, and poses a substantial challenge to public health [3]. The annual economic loss due to bovine mastitis is estimated to be $147 per cow, particularly because of milk quantity losses and culling [4]. Although the etiology and resistance profile of bacteria causing bovine mastitis have been well documented worldwide, these studies are still limited in Thailand, and the current status of antimicrobial resistance directed toward bacterial genera such as Enterobacterales, *Staphylococcus*, and *Streptococcus* remains unclear [5].

Antibiotics are the first line of defense for the treatment of mastitis [6]. Bacterial pathogens are the major cause (70%) of this disease, followed by non-infectious causes (30%), such as physical trauma and mechanical injuries to the mammary glands [7]. The most common bacterial pathogens contributing to mastitis are streptococci, *Escherichia coli*, *Klebsiella*, and staphylococci species [8, 9]. The prevalence of bovine mastitis is different in various countries, such as Cameroon (34.88%) [10], India (37%) [11], Algeria (37.66%) [12], Ethiopia (39.67%) [13], and Kenya (80%) [14]. In Thailand, the prevalence of bovine mastitis is highly variable among different regions, ranging from 5.35% to 59% [15–17]. Most antibiotics have been reported ineffective due to their widespread use in livestock and humans. These extensive uses could induce mutations in bacterial pathogens, leading them to survive and propagate as antimicrobial-resistant strains [18]. The bacteria can be multidrug-resistant (MDR) and carry antimicrobial-resistant genes to resist different antimicrobial classes, such as β-lactams and tetracyclines (TETs), which are normally used routinely for treating bacterial infections in humans and animals [19]. Thus, having prevalence data on mastitis and information on antimicrobial-resistant bovine mastitis bacteria and understanding the antibiotic-resistant pattern in bacteria causing bovine mastitis are valuable for farmers to select appropriate therapeutic measures and to develop an effective infection control strategy [18].

This study aims to explore bovine mastitis bacterial pathogens in Sakon Nakon, Thailand, and to characterize their antimicrobial susceptibility.

## Materials and Methods

### Ethical approval

This study was approved by the Kasetsart University Institutional Animal Care and Use Committee (ACKU66-ETC-006).

### Study period and location

This study was conducted from June to August 2023 in Sakon Nakhon, Thailand.

### Sampling and sample collection

A total of 674 lactating cows were used from 35 farms. Most dairy cows in this region are crossbred Holstein and raised in tied stalls and milked by a milking bucket-type machine twice a day (morning and afternoon) that produces approximately 8–12 kg of milk/cow/day in all daily farms. The cows were mainly fed by fresh grass, rice straw, and brans. Most dairy farms are small-holder dairy farms with 6–40 milking cows. All daily cows (674) on 35 farms were hand-milked under aseptic conditions and tested with the California Mastitis Test (CMT) [20], and 84 aseptically collected positive samples were collected, placed in sterile tubes, and transported to the laboratory for microbiological analysis within 12 h on ice. Antimicrobials used on daily farms were inquired and recorded in 35 farms.

They were cultured in CHROM agar™ (Paris, France) StrepB, MacConkey agar, and Mannitol salt agar to identify β-hemolytic *Streptococcus*s pp., Enterobacterales, and *Staphylococcus* spp., respectively. All culture media were incubated at 37°C for 72 h, with readings taken every 24 h.

### Bacterial identification

Five to ten colonies from each medium were subjected to the DNA extraction method as described by Barbosa *et al*. [21]. *Streptococcus agalactiae*, *Streptococcus uberis*, *Staphylococcus epidermidis*, *E. coli*, and *Klebsiella pneumoniae* complex (KpnC) (*K. pneumoniae*, *Klebsiella variicola*, and *Klebsiella quasipneumoniae*) were identified using multiplex polymerase chain reaction (PCR) as described in the Supplementary Data (Tables-S1–S4). DNA samples from other bacterial species were amplified for the *sod*A gene using the primers *sod*A-F (5´-CCITAYICITAYGAYGCIYTIGARCC-3´) and *sod*A-R (5´-ARRTARTAIGCRT GYTCCCAIACRTC-3´). The PCR program consisted of initiating at 95°C for 3 min, followed by 35 cycles of amplification, denaturation at 95°C for 30 s, annealing at 37°C for 60 s, and elongation at 72°C for 45 s [22]. The PCR products of *sod*A were subjected to Sanger DNA sequencing for species confirmation.

### Detection of antimicrobial resistance genes

Antimicrobial-resistant genes for TET (*tet*A, *tet*E, *tet*G, *tet*K, *tet*L, *tet*M, *tet*O, and *tet*S) and macrolide (*erm*(B), *mef*(A), and *msr*(D)) of *S. agalactiae* and *S. uberis* were analyzed using PCR (Supplementary data). The antimicrobial-resistant genes of *E. coli* and KpnC were detected for the plasmid-mediated quinolone resistance genes for quinolone (Supplementary data), β-lactamase genes (*bla*CTX-M, *bla*TEM, and *bla*SHV) (Supplementary data), CTX-M groups (Supplementary data), carbapenemase genes (*bla*IMP, *bla*KPC, *bla*VIM, *bla*NDM, and *bla*OXA-48-like) (Supplementary data), and the mobile colistin resistance genes (*mcr-1–mcr-9*) (Supplementary data). *mec*A and *mec*C genes of *Staphylococcus* spp. were determined using PCR (Supplementary data).

### Antimicrobial susceptibility

Antimicrobial resistance testing was performed and interpreted according to the recommendations of the Clinical and Laboratory Standards Institute, 2022 [23]. All *S. agalactiae*, *S. uberis*, *S. epidermidis*, *S. aureus*, and Enterobacterales (*E. coli* and KpnC) isolates were investigated for their antimicrobial susceptibility using the microdilution method or disk diffusion, as described in the Supplementary Data (Supplementary data). The antimicrobials used were clindamycin (CLI) (2 μg), chloramphenicol (CHL) (30 μg), TET (30 μg), levofloxacin (LFX) (5 μg), ceftriaxone (CRO) (30 μg), cefoxitin (30 μg), ceftazidime (30 μg), ciprofloxacin (5 μg), and meropenem (10 μg) by disk diffusion (Supplementary data). TET, LFX, erythromycin (ERY), CLI, and penicillin (PEN) were tested by broth microdilution (Supplementary data).

## Results

### Grading of bovine mastitis based on CMT and clinical examination

A total of 35 dairy farms were screened for clinical and subclinical mastitis in Sakon Nakhon, Thailand. Of them, 13 (37.14%) and 30 (85.71%) farms were positive for subclinical and clinical mastitis, respectively. The prevalence of bovine mastitis per cow was categorized into clinical (23.81%, 20/84) and subclinical diseases (76.19%, 64/84). Among subclinical mastitis, 7.81% (5/64) animals were weakly positive (+1), 56.25% (36/64) were distinctly positive (+2), and 35.94% (23/64) were strongly positive (+3). All clinical mastitis cases (100%, 20/20) were strongly positive (+3) for CMT.

### Prevalence of bacteria

Table-1 shows that PCR and DNA sequencing identified *S. epidermidis* (38.10%; 32/84), *S. agalactiae* (33.33%; 28/84), *S. uberis* (25%, 21/84), *K. pneumoniae* (8.33%; 7/84), *S. aureus* (4.76%, 4/84), *Streptococcus hyovaginalis* (4.46%, 4/84), *Streptococcus henryi* (4.46%, 4/84), *Streptococcus gallolyticus* (2.38%, 2/84), *K. variicola* (2.38%, 2/84), *K. quasipneumoniae* (1.19%; 1/84), *E. coli* (1.19%; 1/84), *S. pluranimalium* (1.19%; 1/84), and *E. faecalis* (1.19%; 1/84).

**Table-1:** Distribution of bacterial pathogens isolated from mastitis in dairy cows.

No.	Bacterial species	Cows (n = 84)	Prevalence (%)
1	*Staphylococcus epidermidis*	32	38.10
2	*Streptococcus agalactiae*	28	33.33
3	*Streptococcus uberis*	21	25
4	*Klebsiella pneumonia*	7	8.33
5	*Staphylococcus aureus*	4	4.76
6	*Streptococcus acidominimus/hyovaginalis*	4	4.76
7	*Streptococcus henryi*	4	4.76
8	*Streptococcus gallolyticus*	2	2.38
9	*Klebsiella variicola*	2	2.38
10	*Klebsiella quasipneumoniae*	1	1.19
11	*Escherichia coli*	1	1.19
12	*Streptococcus pluranimalium*	1	1.19
13	*Enterococcus faecalis*	1	1.19

### Distribution of antimicrobial resistance genes

The antimicrobial-resistant genes of *S. agalactiae* revealed that *tet*M was predominant in 68.91% (51/74), followed by *tet*M + *msr*(D) (6.75%, 5/74) and *tet*S + *tet*M + *erm*(B) (1.35%, 1/74) (Table-2).

**Table-2:** Distribution of tetracycline and macrolide-resistant genes in *S. agalactiae* and *S. uberis* isolates from mastitis in dairy cows in Thailand.

Isolates	Tetracycline resistant genes (%)		Macrolide resistance genes (%)			Tetracycline + Macrolide resistance genes (%)			
		
	*tet* (M)	*tet* (S)	*erm* (B)	*mef* (A)	*msr* (D)	*tet* (M) + *msr* (D)	*tet* (S) + *erm* (B)	*tetM* + *mef* (A)	*tetS* + *tetM* + *erm* (B)
*S. agalactiae* (n = 74)	51 (68.91)	-	-	-	-	5 (6.75)	-	-	1 (1.35)
*S. uberis* (n = 68)	28 (41.18)	6 (8.82)	3 (4.41)	-	1 (1.47)	-	24 (35.29)	1 (1.47)	-
Total n = 142	79 (55.63)	6 (4.22)	3 (2.11)	-	1 (0.70)	5 (3.52)	24 (16.90)	1 (0.70)	1 (0.70)

*S. agalactiae=Streptococcus agalactiae, S. uberis=Streptococcus uberis*

*S. uberis* isolates harbored the TET and macrolide-resistant genes. The *tetM* gene was identified in 41.18% (28/68) of all isolates, followed by the *tet*S + *erm*(B) (35.29%, 24/68), *tet*S (8.82%, 6/68), *erm*(B) (4.41%, 3/68), *msr*(D) (1.47%, 1/68), and *tet*M + *mef*(A) (1.47%, 1/68) (Table-2).

The β-lactamase genes in the *E. coli* and KpnC revealed that the *bla*SHV was predominant in *K. pneumoniae* (81.82%, 18/22), followed by *K. variicola* (25%, 1/4). The β-lactamase gene *bla*TEM was mainly identified in *K. pneumoniae* (13.64%, 3/22) and *K. quasipneumoniae* (25%, 1/4). Coexisting *bla*TEM + *bla*CTX-M-9 genes were found in *E. coli* (100%, 4/4) (Table-3). The *oqx*AB gene was identified in *K. pneumoniae* (72.73%, 16/22) and *K. variicola* (50%, 2/4). Coexisting *oqx*AB + *qnr*S genes were present in *E. coli* (100%, 4/4) and *K. pneumoniae* (9.09%, 2/22) (Table-4). This study detected no carbapenemase or colistin-resistant genes in *E. coli* and KpnC isolates. The *mec*A and *mec*C genes were not detected in any of the *S. epidermidis* or *S. aureus* isolates.

**Table-3:** Distribution of β-lactamases genes in *E. coli* and *K. pneumoniae* complex species isolated from bovine mastitis in Thailand.

Isolates (n = 34)	β-lactamases genes (%)			

	*bla*TEM	*bla*SHV	*bla*CTX-M	*bla*TEM + *bla*CTX-M-9
*K. pneumoniae* (n = 22)	3 (13.64)	18 (81.82)	-	-
*K. quasipneumoniae* (4)	1 (25)	-	-	-
*K. variicola* (4)	-	1 (25)	-	-
*E. coli* (n = 4)	-	-	-	4 (100)

*K. pneumoniae=Klebsiella pneumoniae, K. quasipneumoniae=Klebsiella quasipneumoniae, K. variicola=Klebsiella variicola, E. coli=Escherichia coli*

**Table-4:** Distribution of PMQR in *E. coli* and *K. pneumoniae* complex isolated from bovine mastitis in Thailand.

Isolates (n = 34)	PMQR (%)					

	*qnrA*	*aac (6’)-Ib-cr*	*oqx*AB	*qnr*S	*qnr*B	*oqx*AB + *qnr*S
*K. pneumoniae* (n = 22)	-	-	16 (72.73)	-	-	2 (9.09)
*K. quasipneumoniae* (4)	-	-	-	-	-	-
*K. variicola* (4)			2 (50)	-	-	-
*E. coli* (n = 4)	-	-	-	-	-	4 (100)

*K. pneumoniae=Klebsiella pneumoniae, K. quasipneumoniae=Klebsiella quasipneumoniae, K. variicola=Klebsiella variicola, E. coli=Escherichia coli*, PMQR=Plasmid-mediated quinolone resistance

### Antimicrobial susceptibility

**Figure-1:**
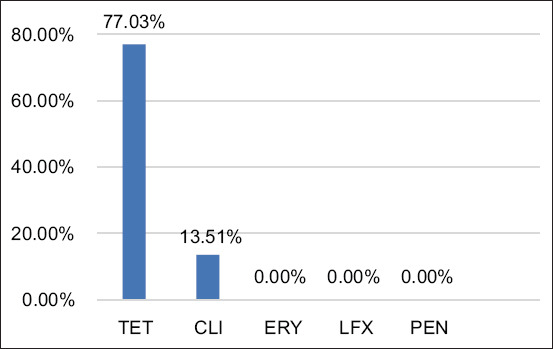
Antimicrobial resistance profiles of Streptococcus agalactiae. TET=Tetracycline, CLI=Clindamycin, ERY=Erythromycin, LFX=Levofloxacin, PEN=Penicillin.

Figure-1 shows the resistance pattern of *S. agalactiae* isolates against five antibiotics. *S. agalactiae* were resisted to TET (77.03%; 57/74) and CLI (13.51%; 10/74) and intermediated susceptibility to ERY (4.05%; 3/74). However, all *S. agalactiae* isolates were susceptible (100%; 74/74) to PEN and LFX (Figure-1).

**Figure-2:**
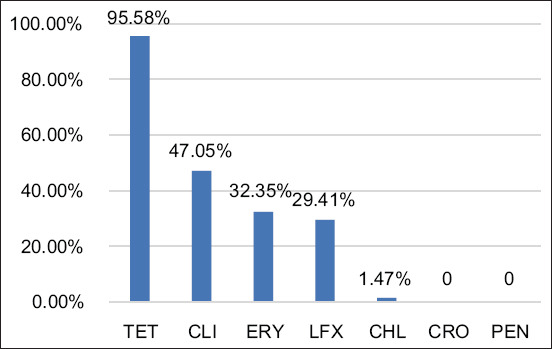
Antimicrobial resistance profiles of Streptococcus uberis. TET=Tetracycline, CLI=Clindamycin, ERY=Erythromycin, LFX=Levofloxacin, CHL=Chloramphenicol, CRO=Ceftriaxone, PEN=Penicillin.

*S. uberis* isolates were resistant to TET (95.58%, 65/68), CLI (47.05%, 32/68), LFX (29.41%, 20/68), CHL (1.47%, 1/68), and ERY (32.35%, 22/68). However, these isolates showed 100% susceptibility to CRO and PEN (Figure-2).

*K. pneumoniae* was susceptible to meropenem (100%, 22/22), followed by ceftazidime (95.45%, 21/22), CRO (86.36%, 19/22), and ciprofloxacin (86.36%, 19/22) (Table-5). All *K. quasipneumoniae* (4/4) and *K. variicola* (4/4) isolates were susceptible to meropenem, ceftazidime, CRO, and ciprofloxacin (Table-5). *E. coli* were also susceptible to ceftazidime (100%, 4/4) and meropenem (100%, 4/4) but 100% resistant to CRO (4/4) and ciprofloxacin (4/4) (Table-5). *S. epidermidis* (100%, 111/111) and *S. aureus* (100%, 41/41) were susceptible to cefoxitin.

**Table-5:** Antimicrobial susceptibility of enterobacteria species isolated from bovine mastitis in Thailand.

Isolates (n = 34)	No. of susceptibility (%)			

	Meropenem	Ceftazidime	Ceftriaxone	Ciprofloxacin
*K. pneumoniae* (n = 22)	22 (100)	21 (95.45)	19 (86.36)	19 (86.36)
*K. quasipneumoniae* (n = 4)	4 (100)	4 (100)	4 (100)	4 (100)
*K. variicola* (n = 4)	4 (100)	4 (100)	4 (100)	4 (100)
*E. coli* (n = 4)	4 (100)	4 (100)	0	0

*K. pneumoniae=Klebsiella pneumoniae, K. quasipneumoniae=Klebsiella quasipneumoniae, K. variicola=Klebsiella variicola, E. coli=Escherichia coli*

### Antimicrobials used on daily farms

**Figure-3:**
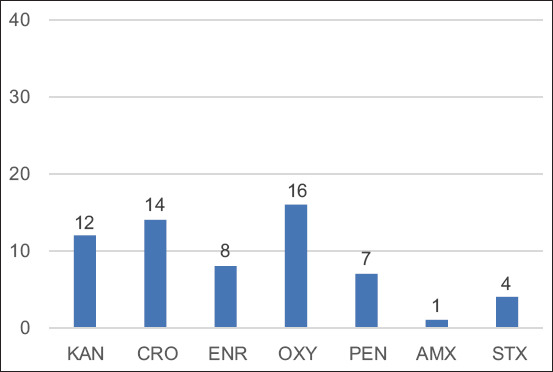
Antimicrobials used in dairy farms for treating clinical and subclinical mastitis. KAN=Kanamycin, CRO=Ceftriaxone, ENR=Enrofloxacin, OXY=Oxytetracycline, PEN=Penicillin, AMX=Amoxicillin, STX=Sulfamethoxazole-trimethoprim.

Eight different types of antimicrobials were used in 35 farms. The highest antimicrobials used for the treatment of bovine mastitis were oxytetracycline in 16 farms (45.71%), CRO in 14 (40%), and kanamycin in 12 (34.29%), as summarized in Figure-3. The frequency of use of enrofloxacin in eight farms (22.86%), PEN in seven (20%), sulfamethoxazole-trimethoprim in four (11.43%), and amoxicillin in one (2.86%) was minimal (Figure-3). The farms used three antimicrobials (17.14%) for bovine mastitis treatment.

## Discussion

In this study, the overall prevalence of bovine mastitis was 12.46% (84/674), which mainly found subclinical mastitis (76.19%) more than clinical mastitis (23.81%). This finding is consistent with previous reports in Kenya (73.1%) [14], Ethiopia (76%) [24], and Rwanda (76.2%) [25]. The higher prevalence of subclinical mastitis (76.19%) than clinical mastitis (23.81%) could be associated with clinical mastitis being easy to diagnose and treat [26], whereas no physical abnormalities and clinical signs of the subclinical form lead to challenges for farmers to diagnose [24, 27].

Our study reported the presence of predominant Gram-positive cocci of *S. agalactiae* (33.33%), *S. uberis* (25%), and *S. aureus* (4.76%) isolated from bovine mastitis. In Brazil, *S. agalactiae* was predominantly identified in the bacterial genus *Streptococcus* in bovine mastitis [28]. However, several studies have reported that *S. uberis* is the most common bacterial cause of recurrent bovine mastitis with clinical and subclinical infections worldwide [19, 29–31]. Our study reported *S. uberis* resistance to TET (95.58%, 65/68), CLI (47.05%, 32/68), LFX (29.41%, 20/68), and CHL (1.47%, 1/68). Consistent with previous studies by Abd El-Aziz *et al*. [32], *S. uberis* isolated from bovine mastitis in Egypt was resistant to TET (65.22%), CLI (100%), CHL (55.07%), and CRO (100%). From 2010 to 2017, Zhang *et al*. [33] reported that most *S. uberis* strains associated with bovine mastitis in northern Thailand were resistant to TET (82.02%), followed by ceftiofur (cephalosporins) (19.30%) and ERY (8.33%). Similarly, Zhang *et al*. [34] demonstrated an increasing trend of CRO-resistant strains of *Streptococcus dysgalactiae* associated with bovine mastitis in China. However, our study found that this pathogen was still susceptible to CRO (100%).

Among the *S. uberis* isolates, the most TET - and macrolide-resistant genes are the *tet*M gene (41.18%), followed by the *tet*S + *erm*(B) (35.29%), *tet*S (8.82%), *erm*(B) (4.41%), *msr*(D) (1.47%), and *tet*M + *mef*(A) (1.47%). Consistent with previous reports by Kaczorek *et al*. [19] and Zhang *et al*. [33], the most common genes detected in *S. uberis* isolates was *tet*M in Poland (64%) and Thailand (87.28%). However, the *erm*(B) gene is the predominant antimicrobial-resistant gene (75.36%) in *S. uberis* isolated from clinical mastitis in dairy cows in Egypt [32].

TET is a broad-spectrum antimicrobial agent administered to cows with mastitis for clinical recovery of infection [35]. TET showed 45.71% use for the treatment of bovine mastitis in this study. Phenotypically resistant isolates in *S. agalactiae* were resistant to TET (77.03%; 57/74) and CLI (13.51%; 10/74) and intermediated to ERY (4.05%; 3/74), and all isolates were susceptible to PEN and LFX.

A previous study by De Oliveira *et al*. [28] revealed that bovine mastitis *S. agalactiae* was resistant to TET (75.9%), followed by ampicillin and PEN (56.2%). The antimicrobial-resistant profiles of *S. agalactiae* in this study showed a high resistance rate to TET, similar to a previous study by Leghari *et al*. [18], which could be associated with the extensive use of this antibiotic in treatment, even prophylaxis, or as a component in feed [36]. The antimicrobial-resistant mechanism to TET of this species is ribosomal protection due to the high prevalence of *tet*M and other *tet* family genes [28, 37]. The genotypic profiles of *S. agalactiae* species were identified in 68.91% of all isolates that were positive for *tet*M gene, followed by 6.75% for *tet*M + *msr*(D) and 1.35% for *tet*S + *tet*M + *erm*(B). Previously, the *tet*O gene was predominant in this pathogen cause bovine mastitis in China [18], Pakistan [18], and the USA [38]. Most *S. agalactiae* isolates were identified for *tet*M (43%), followed by *tet*L (31.9%) and *bla*Z (26.3%) [28]. In China, *S. agalactiae* isolated from mastitis milk samples were mainly *tet*M (46.67%), followed by *tet*K (40%), *tet*S (40%), and *tet*O (33.33%) [39].

Our findings suggest that bovine mastitis caused by *S. agalactiae* and *S. uberis* should be treated with antimicrobials other than TET, such as beta-lactams and fluoroquinolones, to prevent the further spread of TET-resistant clones. Similarly, several studies have reported that β-lactams were effective against *Streptococcus* isolates [18, 29, 40]. Thus, PEN was still an effective treatment for this infection. However, there is a need for improvement in sanitation practices, such as enhanced pre- and post-milking hygiene and effective treatment with suitable antibiotics. Unfortunately, the widespread and incorrect use of antibiotics has increased, leading to the emergence of antibiotic-resistant pathogens.

In our study, the highest frequency of *S. epidermidis* (38.10%) in bovine mastitis in northeast Thailand was observed. *S. epidermidis* is one of the frequently isolated species in subclinical mastitis but can be a persistent infection in many countries [41, 42] and associated with decreased milk quality [43]. However, the distribution of staphylococci and streptococci in milk samples of mastitis cows and buffaloes in India was *S. aureus*, *S. epidermidis*, *S. agalactiae*, *S. uberis*, and *S. dysgalactiae* as 64.9%, 7.7%, 48.7%, 65.8%, and 0.8%, respectively [44]. *S. epidermidis* is absent or very rare in the normal bovine skin flora or mucous membrane flora [45, 46]. A previous study by Watts and Owens [47] and Thorberg *et al*. [48] reported that udder infections in bovines caused by *S. epidermidis* originated from farmers who transferred these bacteria during daily contact with the udders. Thus, post-milking hygiene or milking equipment may effectively reduce *S. epidermidis* infection [41, 43].

In this study, Gram-negative bacterial isolates (*K. pneumoniae*, *K. variicola*, *K. quasipneumoniae*, and *E. coli*) were recovered from clinical and subclinical mastitis patients. The predominant bacterial species was *K. pneumoniae*. Gram-negative bacteria causing bovine mastitis are classified as environmental pathogens [49]. The antimicrobial resistance of Gram-negative bacteria isolated from bovine mastitis has been reported in many countries [50–52]. Among Gram-negative bovine mastitis pathogens, *E. coli* was mainly isolated from bovine mastitis milk [30, 53]. Previously, 90.7% of *E. coli* isolated from dairy cows with mastitis were MDR in the USA [54]. Lehtolainen *et al*. [55] reported that 11% of *E. coli* isolated from clinical bovine mastitis were MDR types in Finland and Israel. However, Gram-negative bacterial isolates causing bovine mastitis in our study were still sensitive to meropenem (100%), ceftazidime (97.06%), CRO (79.41%), and ciprofloxacin (79.41%). This study identified the β-lactamase genes *bla*SHV in 70.37% (19/27) of Gram-negative bacterial isolates. In addition, *bla*TEM, a narrow-spectrum β-lactamase gene, which confers resistance to PENs and first-generation cephalosporins, was identified as 14.81%. Furthermore, co-harboring of *bla*TEM and the extended-spectrum β-lactamase-encoding genes *bla*CTX-9 were also identified. The *bla*CTX gene is the most prevalent gene in Enterobacterales isolated from bovine mastitis milk in other countries [53, 56].

## Conclusion

*S. epidermidis* exhibited the highest prevalence as a causative agent of bovine mastitis in Sakon Nakhon, Thailand. Meanwhile, *Streptococcus* genus, such as *S. agalactiae* and *S. uberis*, demonstrated resistance to TET and mainly harbored *tet* family genes. *K. pneumoniae* isolates were susceptible to some antibiotics. This study suggests that periodic surveillance of antibiotic susceptibilities and molecular characterization of pathogenic bacteria isolated from mastitis cows are important measures for detecting the emergence and spread of antimicrobial-resistant isolates. In this study, limited data were available including one from small-holder dairy farms and study only dairy farms in Sakon Nakhon, Thailand. So, more farms should be included in the future studies.

## Data Availability

The supplementary data can be available from the corresponding author on a reasonable request.

## Authors’ Contributions

PB: Conceptualization and design of the study and drafted the manuscript. AC: Performed the laboratory work. AC, NP, and BP: Sample collection. PB, PP, NW, SY, PC, and AK: Performed validation and data analysis. All authors have read, reviewed, and approved the final manuscript.
